# Case Report: From chronic infection to disseminated strongyloidiasis: a case of corticosteroid-induced hyperinfection syndrome

**DOI:** 10.3389/fimmu.2026.1855927

**Published:** 2026-07-16

**Authors:** Yingying Lu, Jiaqi Su, Yinping You, Jinyan Kai

**Affiliations:** 1 Department of Clinical Laboratory, The Second Affiliated Hospital of Xiamen Medical College, Xiamen, China; 2 Department of Pathology, The Second Affiliated Hospital of Xiamen Medical College, Xiamen, China

**Keywords:** corticosteroid, disseminated infection, eosinophil, hyperinfection syndrome, *Strongyloides stercoralis*

## Abstract

**Background:**

*Strongyloides stercoralis* (*S. stercoralis*) is a soil-transmitted nematode predominantly endemic to tropical and subtropical regions, capable of causing life-threatening hyperinfection syndrome and disseminated infection in immunocompromised individuals. With the improvement of sanitation conditions, strongyloidiasis has become increasingly uncommon. Early-stage infection presents with mild, nonspecific symptoms, posing diagnostic challenges.

**Case description:**

We report a case of *S. stercoralis* infection in a 73-year-old female patient, evolving from initial erythematous rash to disseminated disease. The patient, with a 30-year history of bronchial asthma, developed severe hyperinfection syndrome following corticosteroid therapy. She initially presented with cutaneous petechiae and pruritus, and was diagnosed with allergic purpura. With exacerbation of respiratory symptoms, she was diagnosed with asthma complicated by fungal infection. After treatment with methylprednisolone and itraconazole, severe gastrointestinal symptoms developed. The diagnosis was confirmed only after simultaneous detection of *S. stercoralis* in multiple body fluid specimens. Ultimately, following discontinuation of methylprednisolone and itraconazole, and administration of albendazole, the patient gradually recovered to normal.

**Conclusion:**

This case highlights the diagnostic challenges in the early stages of *S. stercoralis* infection and demonstrates the characteristic dissociation between leukocytosis and eosinopenia during corticosteroid-induced hyperinfection syndrome. Prompt recognition and immediate discontinuation of immunosuppressive therapy combined with anthelmintic treatment are critical for favorable outcomes in disseminated strongyloidiasis.

## 1 Introduction

Strongyloidiasis is an opportunistic parasitic disease caused by infection with *Strongyloides stercoralis* (*S. stercoralis*), predominantly endemic to tropical and subtropical regions (1). Humans serve as the principal definitive host for the adult worms of the parasitic generation. Eggs hatch into rhabditiform larvae within hours in warm, moist soil. Following two molts, rhabditiform larvae develop into filariform larvae, which are infective to the host and can penetrate human skin or mucous membranes. Adult worms primarily parasitize the small intestine of the host (2). Chronic strongyloidiasis is typically asymptomatic or manifests solely as eosinophilia. Some cases may present with mild gastrointestinal or cutaneous symptoms (3). In immunocompromised hosts, the disease readily progresses to hyperinfection syndrome and disseminated infection, with high mortality (4).

Herein, we report the case of a 73-year-old female with a 30-year history of bronchial asthma who developed corticosteroid-induced hyperinfection syndrome progressing to disseminated strongyloidiasis. This case illustrates several important clinical lessons: (i) the necessity of suspecting underlying chronic strongyloidiasis and considering screening before initiating systemic corticosteroids in patients with epidemiological risk factors; (ii) the nonspecific early manifestations may mimic allergic purpura or fungal infection, leading to diagnostic delay; and (iii) the diagnostic value of multi-site and repeated sampling once dissemination occurs. By presenting this complete clinical trajectory, we aim to emphasize that a high index of suspicion and timely discontinuation of immunosuppression are essential to prevent catastrophic outcomes in patients with undiagnosed chronic strongyloidiasis.

## 2 Case presentation

A 73-year-old female patient presented to the dermatology department on March 28, 2025, with a one-month history of petechiae and ecchymoses on the abdomen and lower extremities, accompanied by pruritus. She had a 30-year history of cough with occasional white sputum, dyspnea, and chest tightness exacerbated by exertion, diagnosed as bronchial asthma in 2017. Notably, she had a habit of scavenging through refuse for over 10 years, with substantial soil and dust exposure.

Initial misdiagnosis and corticosteroid exposure. Given markedly elevated IgE (1476 KIU/L), normal eosinophil count, and refusal of skin biopsy, allergic purpura was diagnosed and treated with antihistamines. On April 8, 2025, she was admitted to the respiratory department due to aggravated cough and dyspnea. *Aspergillus* IgM was positive (138.31 AU/mL), while IgG was negative. The diagnosis of asthma with fungal infection was made. Oral itraconazole (0.2 g qd) and intravenous methylprednisolone (40 mg qd) were initiated on April 16. Dyspnea improved and cutaneous rashes regressed. However, when methylprednisolone was tapered to 20 mg on April 22, rashes recurred; re-escalation to 40 mg on April 26 led to regression again. She was discharged on May 1 with continued oral therapy.

**Figure 1:**
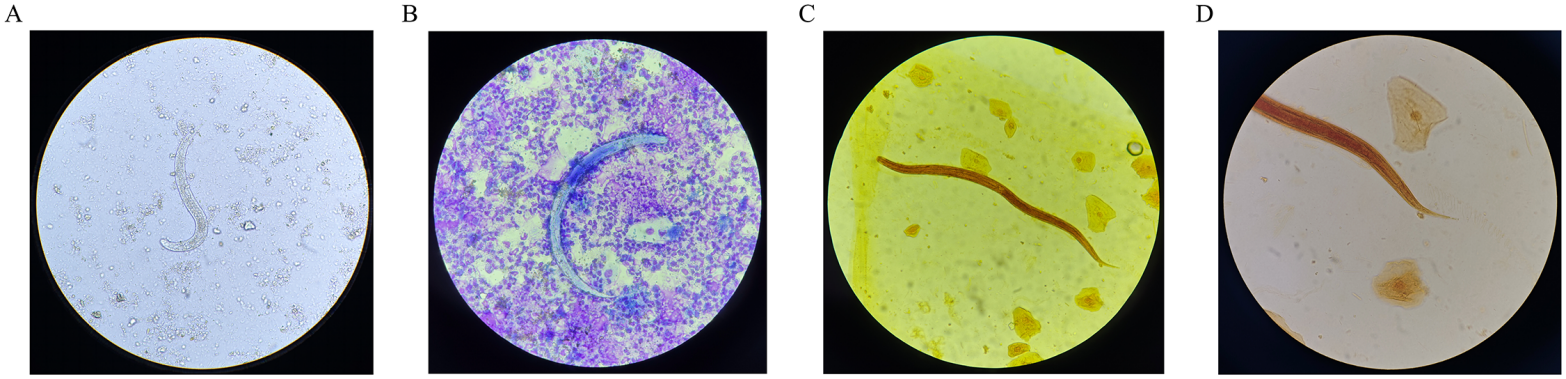
*Strongyloides stercoralis* detected in stool and sputum specimens. **(A)** Rhabditiform larvae of *S. stercoralis* detected in fecal smear (×400); **(B)** Wright-Giemsa stain of sputum revealing *S. stercoralis* with prominent neutrophilic infiltrate (×400); **(C)** Filariform larvae of *S. stercoralis* in Lugol's iodine-stained sputum specimen (×400); **(D)** Bifid tail of filariform larva observed in Lugol's iodine-stained sputum specimen (×1000).

Dissemination and definitive diagnosis. On May 9, severe abdominal pain and watery diarrhea (>10 episodes daily) developed, without fever, vomiting, or hematochezia. She self-discontinued medications and was readmitted on May 12. Blood examination revealed leukopenia (WBC 2.84×10^9^/L) and eosinopenia (1.1%). Notably, despite three prior stool examinations by direct smear (March 28, April 9, and April 15) yielding negative results, nematodes were simultaneously detected in fecal, urinary, and sputum smears on May 13 by clinical laboratory (**Figure 1**; **Supplementary Material 1**). The samples were forwarded to the Xiamen Center for Disease Control and Prevention for species confirmation. Morphological examination of fecal larvae under light microscopy (40× objective) revealed actively motile organisms measuring 0.60–0.70 mm in length, with a cylindrical pharynx, prominent genital primordium in the posterior body, and a pointed, bifurcated tail—features characteristic of *S. stercoralis* filariform larvae (L3). The absence of a bulbous esophagus excluded hookworm larvae, and the presence of a genital primordium distinguished these from *Ancylostoma* or *Necator* species. Other nematode larvae (e.g., *Trichostrongylus*) were excluded based on morphometric and structural criteria. Skin biopsies from the abdomen and thigh performed on May 15 were negative for parasites (**Supplementary Material 2**).

**Figure 2:**
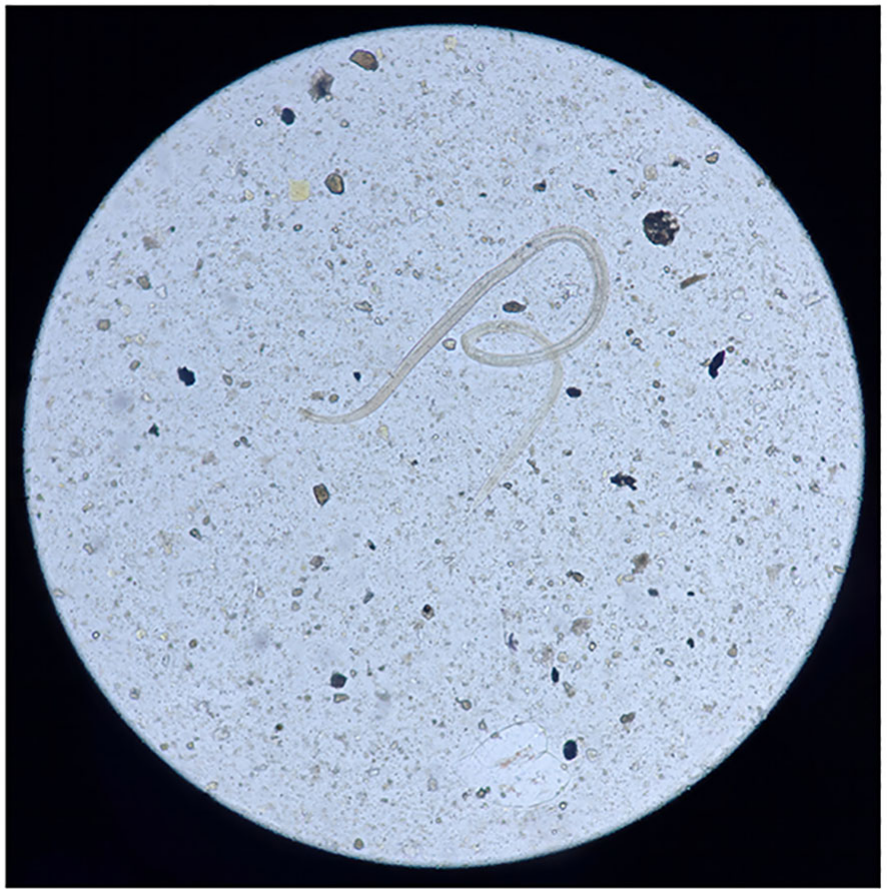
Direct smear of fecal specimen revealed dead adult worms of *S. stercoralis*, indicating the efficacy of anthelmintic therapy.

Treatment response and recovery. Itraconazole and methylprednisolone were discontinued on May 14, and albendazole (0.4 g qd) was initiated. Dead adult worms were observed in fecal smears on May 19 (**Figure 2**). After completing one week of anthelmintic therapy on May 21, with supportive care, her condition gradually improved. She was discharged on June 1 with resolution of all symptoms. Follow-up revealed no recurrence of rashes or asthma symptoms.

**Figure 3:**
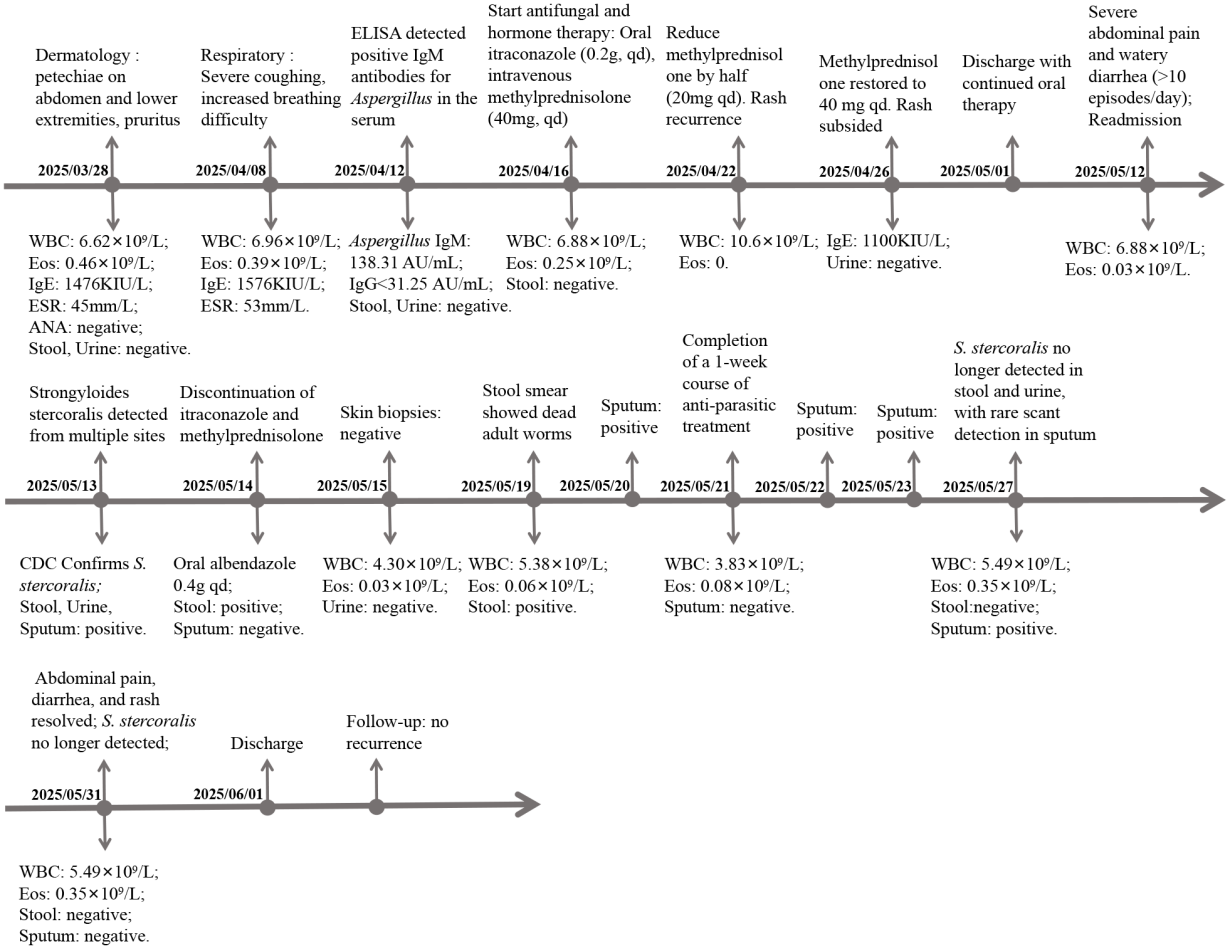
The diagnostic and therapeutic course and monitoring indicators of the patient.

The complete clinical course, laboratory parameters, and treatment timeline are summarized in **Table 1**, **Supplementary Table 1** and **Figure 3**.

**Table 1:** Summary of diagnostic and therapeutic timeline.

Date	Clinical Event	WBC(×10^9^/L)	Eos(×10^9^/L)	Eos%	Other Laboratory Tests	Stool Exam	Urine	Sputum	Microbiology	Treatment	Clinical Outcome
2025.3.28	Dermatology : petechiae on abdomen and lower extremities, pruritus	6.62	0.46	6.9	IgE: 1476KIU/L; ESR: 45mm/h; ANA: Negative	Negative	Negative	—	—	Epinastine, cetirizine, dipyridamole	Symptoms improved
2025.4.8	Respiratory : Severe coughing, increased breathing difficulty	6.96	0.39	5.6	IgE: 1576KIU/L; ESR: 53mm/h	—	—	—	—	—	—
2025.4.12	ELISA detected positive IgM antibodies for *Aspergillus* in the serum	—	—	—	*Aspergillus* IgM: 138.31 AU/mL; IgG<31.25AU/mL	Negative	—	Negative	Normal respiratory tract flora	—	—
2025.4.16	Start antifungal and hormone therapy	6.88	0.25	3.6	—	Negative	—	—	—	Itraconazole 0.2g qd+ Methylprednisolone 40mg iv qd+ Nebulized bronchodilator	Dyspnea relieved, Rash subsided
2025.4.22	Reduce methylprednisolone by half	10.6	0	0	—	—	—	—	—	Methylprednisolone 20mg qd	Rash recurrence
2025.4.26	Methylprednisolone restored to 40 mg	—	—	—	IgE: 1100KIU/L	—	Negative	—	—	Methylprednisolone 40mg qd	Rash subsided
2025.5.1	Discharge	—	—	—	—	—	—	—	—	Oral itraconazole and methylprednisolone	—
2025.5.12	Severe abdominal pain and watery diarrhea (>10 episodes/day); Readmission	2.84	0.03	1.1	—	—	—	—	—	Discontinuation of medication	—
2025.5.13	*Strongyloides stercoralis* detected from multiple sites	—	—	—	—	Positive	Positive	Positive	CDC Confirms *S. stercoralis*	—	Disseminated strongyloidiasis
2025.5.14	Discontinuation of itraconazole and methylprednisolone	2.7	0.01	0.3	—	Positive	—	Negative	—	Albendazole 0.4g qd	—
2025.5.15	Skin biopsies of the abdomen and thigh	4.3	0.03	0.8	—	—	Negative	—	Pathology result:negative for parasites	—	—
2025.5.19	Stool smear showed dead adult worms	5.38	0.06	1.2	—	Positive	—	—	—	Albendazole treatment in progress	—
2025.5.21	Completion of a 1-week course of anti-parasitic treatment	3.83	0.08	2.2	—	—	—	Negative	Acid-fast bacilli smear: negative	Albendazole course completed	—
2025.5.27	*S.* *stercoralis* no longer detected in stool and urine, with rare scant detection in sputum	5.49	0.35	6.4	—	Negative	—	Positive	—	—	Clinical improvement
2025.5.31	*S.* *stercoralis* no longer detected	9.38	0.74	7.9	—	Negative	—	Negative	—	—	Abdominal pain, diarrhea, and rash resolved
2025.6.1	Discharge	—	—	—	—	—	—	—	—	—	—
Follow-up	No recurrence	—	—	—	—	—	—	—	—	—	—

WBC, white blood cell count; Eos, eosinophil; Eos%, eosinophil percentage; IgE, immunoglobulin E; ESR, erythrocyte sedimentation rate; qd, once daily; iv, intravenous; CDC, Center for Disease Control and Prevention; *S. stercoralis, Strongyloides stercoralis*.

## 3 Discussion

*S. stercoralis* is a soil-transmitted nematode primarily distributed in tropical and subtropical regions, including humid areas of southern China (1). Chronic infection is typically asymptomatic or manifests with mild, non-specific cutaneous or gastrointestinal symptoms that are frequently overlooked (4). In China, severe cases of strongyloidiasis still occur occasionally, particularly in immunocompromised populations (5–7). A recent systematic review of 16 cases of hyperinfection and disseminated strongyloidiasis with thrombotic complications reported a mortality of 31.3%, with corticosteroid-induced immunosuppression identified as the predominant risk factor (8). This underscores the catastrophic potential of undiagnosed chronic infection when immunosuppressive therapy is initiated. In non-endemic settings, clinicians frequently fail to include strongyloidiasis in the differential diagnosis of nonspecific gastrointestinal, cutaneous, or respiratory manifestations, leading to dangerous diagnostic delays (9).

This case illustrates the classic progression from chronic *S. stercoralis* infection to corticosteroid-induced hyperinfection syndrome and subsequent dissemination. Its primary value lies in highlighting several critical clinical lessons for practitioners in low-endemicity settings: (i) patients with epidemiological risk factors must undergo repeated multi-site screening to improve diagnosis; (ii) parasitic screening should be considered prior to systemic glucocorticoid use in patients with risk factors or nonspecific manifestations; (iii) immediate discontinuation of immunosuppressive therapy combined with prompt anthelmintic treatment is critical once hyperinfection or dissemination is suspected.

Diagnostic challenges and differential diagnoses. The diagnostic delay in this case exemplifies the challenges of recognizing chronic strongyloidiasis in its early stages. The initial presentation with cutaneous petechiae and markedly elevated IgE (1476 KIU/L), in the context of normal eosinophil counts, led to a provisional diagnosis of allergic purpura. Notably, the subsequent partial response to corticosteroid therapy—initially administered for presumed asthma exacerbation—further reinforced the impression of an allergic or inflammatory etiology, inadvertently masking the underlying parasitic infection (9). This transient clinical improvement likely reflected the anti-inflammatory effects of glucocorticoids on cutaneous larva currens, a hallmark of autoinfection in strongyloidiasis, rather than true resolution of the primary condition. When corticosteroids were tapered, cutaneous manifestations promptly recurred, which in retrospect should have raised suspicion for larva currens rather than simple disease relapse. The opportunity for early histopathological confirmation was lost due to the patient’s refusal of skin biopsy. Subsequently, combined with positive *Aspergillus* IgM and a history of refuse exposure, prompted a diagnosis of asthma with fungal infection. Although the microbiology laboratory failed to find direct evidence of *Aspergillus* infection, antifungal therapy was nonetheless considered as the initial treatment. Notably, the possibility of underlying parasitic infection was not entertained until gastrointestinal deterioration supervened, underscoring how nonspecific manifestations may divert clinical attention in low-prevalence regions.

It is essential to distinguish hyperinfection syndrome from disseminated strongyloidiasis. Hyperinfection syndrome is defined as accelerated autoinfection within the pulmonary-gastrointestinal cycle, with massive larval amplification but confinement to these organs. Disseminated strongyloidiasis, by contrast, involves larval migration to organs outside the usual autoinfective route, including the central nervous system, liver, and urinary tract (10, 11). In this case, detection of larvae in stool and sputum is consistent with hyperinfection. However, simultaneous detection in urine supports dissemination, as the urinary tract is not part of the canonical autoinfective cycle. The urine specimens were clean-catch midstream collections, minimizing fecal contamination. Although larval migration across the compromised urinary mucosa is the most plausible explanation, we acknowledge that complete exclusion of contamination cannot be guaranteed without catheterized specimens.

During corticosteroid therapy, the patient exhibited a characteristic dissociation between rising leukocytosis and declining eosinophil percentage (**Supplementary Material 3**). This phenomenon is clinically relevant because it may mask the eosinophilia typically expected in parasitic infection, thereby contributing to diagnostic delay. Upon readmission, both WBC (2.84×10^9^/L) and eosinophil percentage (1.1%) had fallen below normal ranges. Blood cultures were obtained on May 17 and reported on May 22 yielded no bacterial growth, making significant bacterial translocation unlikely as the primary driver of leukopenia. This cytopenia more likely reflected the combined effects of sustained high-dose glucocorticoid therapy and systemic consumption in the setting of massive parasite proliferation. Following anthelmintic therapy and corticosteroid discontinuation, both parameters gradually recovered. Notably, eosinophil percentage transiently rose to 14.4% during follow-up, possibly reflecting immune-mediated clearance of residual parasites.

The patient received albendazole 400 mg once daily for one week. Ivermectin is generally considered first-line therapy for hyperinfection and disseminated strongyloidiasis due to its superior efficacy and safety profile (11, 12). However, ivermectin was unavailable at our institution at the time of treatment. Albendazole, though less effective, remains an acceptable alternative when ivermectin cannot be obtained. Recent guidelines suggest that in severe or disseminated disease, combination therapy or extended courses may be warranted. We closely followed up the patient’s stool and sputum examinations through July and confirmed no recurrence (**Supplementary Table 1**).

Recent reports have highlighted the severe systemic complications of *S. stercoralis* hyperinfection, including shock, thrombosis, and alveolar hemorrhage (13). López-Delgado et al. described hyperinfection complicated by deep vein thrombosis and pulmonary thromboembolism, emphasizing that corticosteroid-induced immunosuppression is the predominant risk factor (8, 14). While our patient did not develop thrombotic complications, her rapid hematological deterioration underscores the potential for catastrophic progression. These findings reinforce the imperative of early suspicion and prompt antiparasitic therapy before irreversible organ damage occurs.

## 4 Strengths and limitations

The strengths of this report include the detailed chronological documentation of disease progression from chronic infection to disseminated disease, the availability of longitudinal hematological data illustrating glucocorticoid effects, and the confirmation of parasitological clearance through repeated follow-up examinations. However, several limitations must be acknowledged. First, the duration of chronic infection prior to presentation could not be determined. Second, the recurrence of skin symptoms following glucocorticoid tapering failed to raise suspicion of parasitic infection. Third, the diagnosis of fungal infection was based solely on positive Aspergillus IgM, without microbiological evidence to confirm invasive lesions. Fourth, skin biopsies were negative. Fifth, serology, PCR, and agar plate culture were unavailable. Sixth, the possibility of contamination in midstream urine could not be excluded.

## 5 Conclusion

This case demonstrates that parasitic infection screening is necessary for patients with epidemiological risk factors prior to initiating systemic glucocorticoid therapy. In low-prevalence areas, early nonspecific symptoms often mimic common allergic or infectious diseases, leading to diagnostic delay and posing a risk. Once severe infection or dissemination is suspected, immunosuppressive therapy should be discontinued immediately and antiparasitic treatment initiated. When routine test results are negative, multi-site sampling and repeated testing can significantly improve diagnostic accuracy.

## Data Availability

The original contributions presented in the study are included in the article/**Supplementary Material**, further inquiries can be directed to the corresponding author.
