# Supplementary figures and images for: Case Report: From chronic infection to disseminated strongyloidiasis: a case of corticosteroid-induced hyperinfection syndrome
Source: Front Immunol. 2026 Jul 16;17:1855927. doi: 10.3389/fimmu.2026.1855927 (PMC13421396; doi:10.3389/fimmu.2026.1855927)

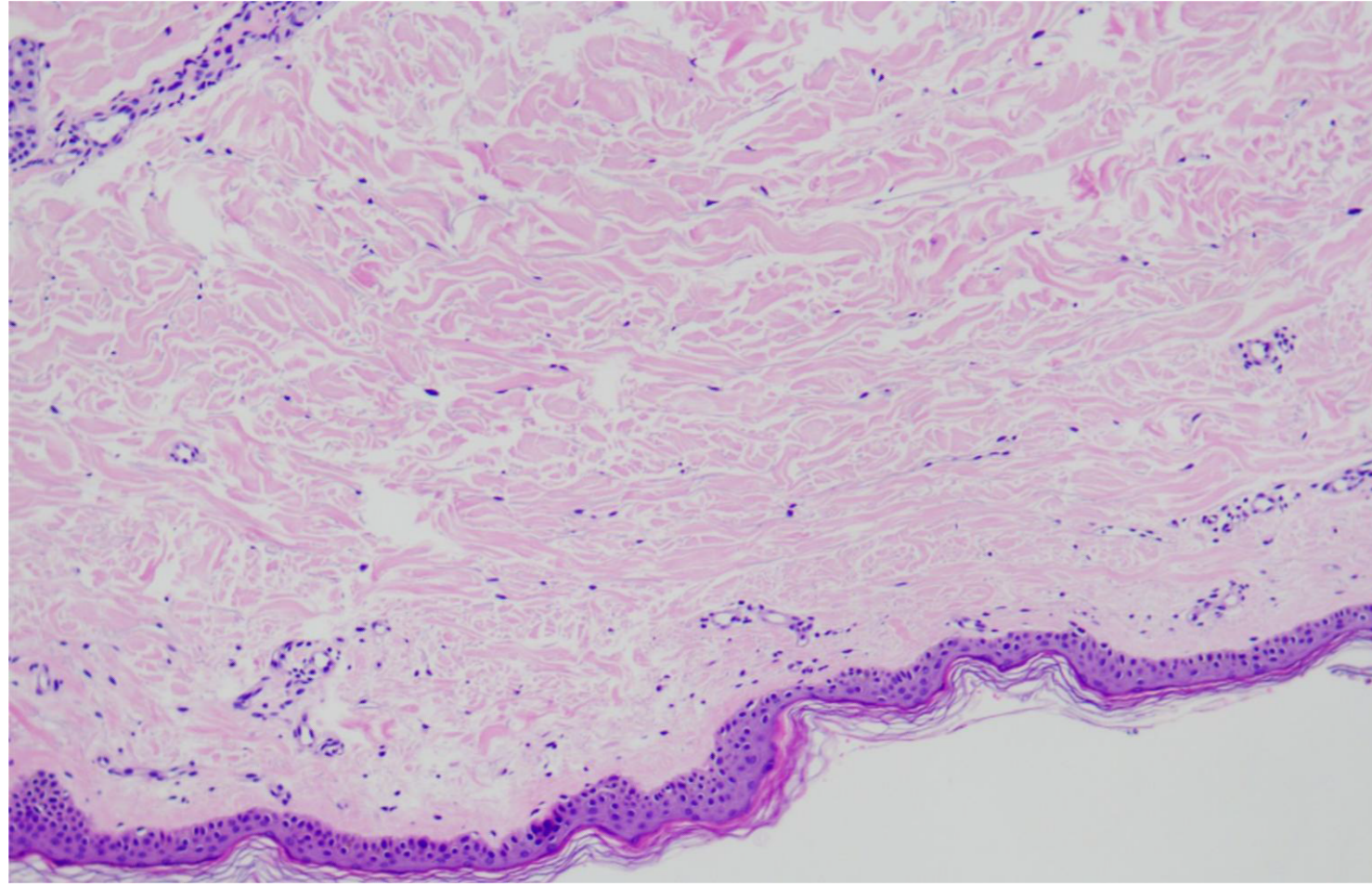

**Figure S2. Pathological HE staining of the dermis layer of the skin.**

Supplement: Supplementary file 1 [file DataSheet1.pdf]

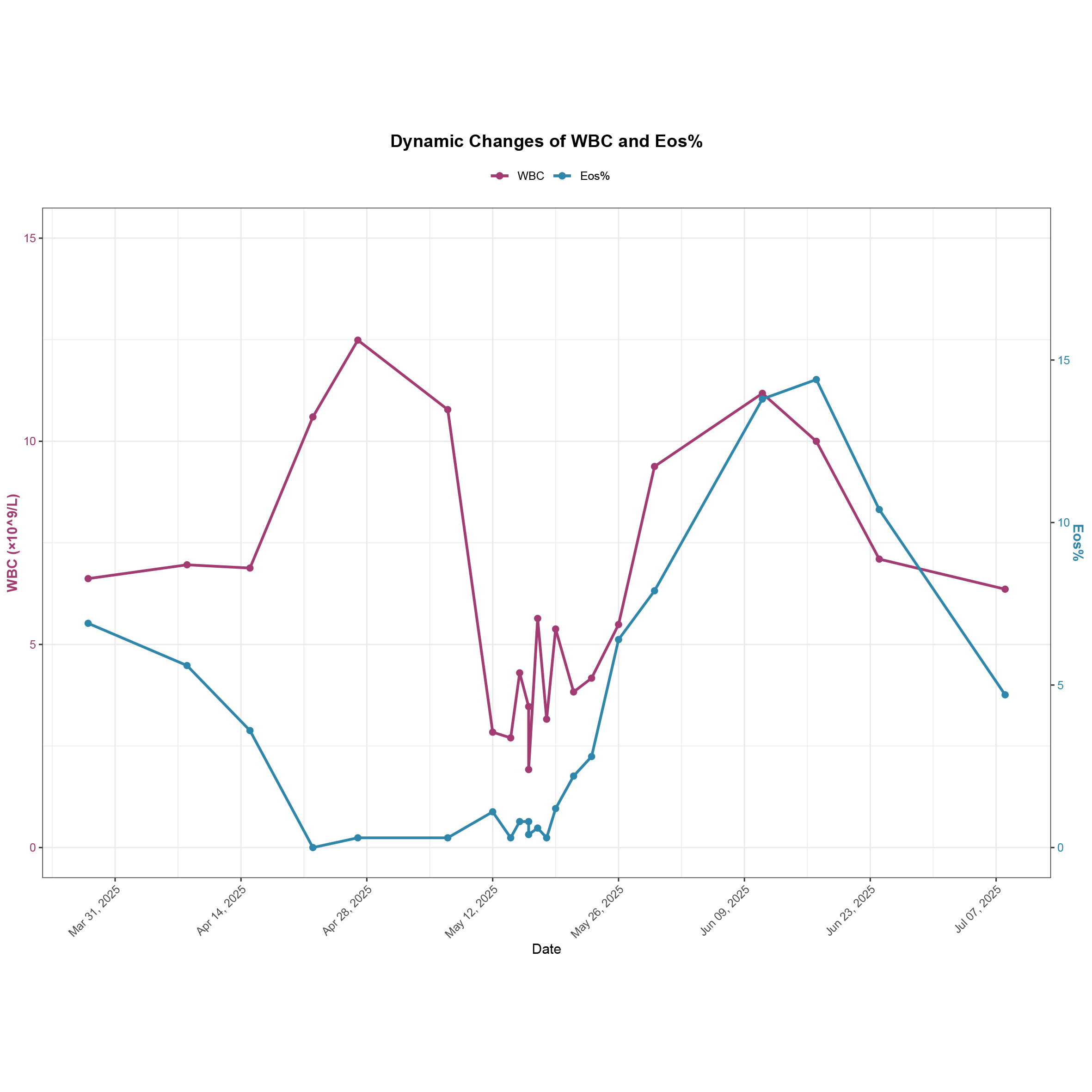

Supplement: Supplementary file 2 [file Image1.tif]
