# Supplementary material for: Case Report: From chronic infection to disseminated strongyloidiasis: a case of corticosteroid-induced hyperinfection syndrome
Source: Front Immunol. 2026 Jul 16;17:1855927. doi: 10.3389/fimmu.2026.1855927 (PMC13421396; doi:10.3389/fimmu.2026.1855927)
Supplement: Supplementary file 3 [file Table1.docx]

**Supplementary** **Table. 1 Detection of *S. stercoralis* in Body Fluid Specimens**

| **Date** | **Stool** | **Urine** | **Sputum** |
| --- | --- | --- | --- |
| 2025/03/28 | **-** | **/** | **/** |
| 2025/03/31 | **/** | **-** | **/** |
| 2025/04/09 | **-** | **/** | **-** |
| 2025/04/11 | **/** | **/** | **-** |
| 2025/04/15 | **-** | **/** | **/** |
| 2025/04/23 | **/** | **-** | **/** |
| 2025/05/13 | **+** | **+** | **+** |
| 2025/05/14 | **+** | **/** | **-** |
| 2025/05/15 | **/** | **-** | **/** |
| 2025/05/17 | **+** | **/** | **/** |
| 2025/05/19 | **+** | **/** | **/** |
| 2025/05/20 | **/** | **/** | **+** |
| 2025/05/21 | **/** | **/** | **-** |
| 2025/05/22 | **-** | **-** | **+** |
| 2025/05/23 | **/** | **/** | **+** |
| 2025/05/24 | **-** | **/** | **/** |
| 2025/05/26 | **/** | **/** | **-** |
| 2025/05/27 | **-** | **/** | **+** |
| 2025/05/29 | **-** | **/** | **/** |
| 2025/05/30 | **/** | **/** | **-** |
| 2025/05/31 | **-** | **/** | **-** |
| 2025/06/11 | **-** | **/** | **/** |
| 2025/06/12 | **/** | **/** | **-** |
| 2025/06/17 | **-** | **/** | **-** |
| 2025/06/24 | **-** | **/** | **-** |
| 2025/07/08 | **-** | **/** | **/** |

+: Positive; -: Negative; /: No specimen received.
